# Exploring the Adaptation Process of *Huso dauricus* to High Temperatures Based on Changes in Intestinal Microbiota

**DOI:** 10.3390/biology13121045

**Published:** 2024-12-13

**Authors:** Ruoyu Wang, Yutao Li, Yining Zhang, Sihan Wang, Zheng He, Dingchen Cao, Zhipeng Sun, Nianmin Wang, Ying Zhang, Bo Ma

**Affiliations:** 1 Key Open Laboratory of Cold Water Fish Germplasm Resources and Breeding of Heilongjiang Province, Heilongjiang River Fisheries Research Institute, Chinese Academy of Fishery Sciences, Harbin 150076, China; wangruoyu@hrfri.ac.cn (R.W.);; 2 College of Fisheries and Life Science, Dalian Ocean University, Dalian 116023, China; 3 College of Fisheries and Life Science, Shanghai Ocean University, Shanghai 201306, China

**Keywords:** high-temperature stress, *Huso dauricus*, intestinal microbiota, high-temperature adaptability

## Abstract

Global warming has led to rising water temperatures, posing a significant threat to fish survival. Understanding the mechanisms by which fish respond to and adapt to temperature variations is thus of considerable importance. This study employed high-throughput 16S rRNA gene sequencing and bioinformatics to investigate changes in the intestinal microbiota of the kaluga sturgeon (*Huso dauricus*) under four temperature conditions (19 °C, 25 °C, 28 °C, and 31 °C) and its relationship with adaptation to high-temperature stress. The results indicated that temperature variations caused significant changes in the intestinal microbiota. Over time, differences in the microbiota structure became more pronounced under different temperature conditions, and within-group variability gradually decreased. At higher temperatures, the relative abundance of *Sphingomonas* significantly decreased, while that of *Clostridium sensu stricto 1*, *Cetobacterium*, and *Plesiomonas* exhibited a significant increase in relative abundance. Upon the cessation of rapid mortality under various high-temperature conditions, the intestinal microbiota structure and composition became highly similar, with *Clostridium sensu stricto 1* dominating both in terms of composition and relative abundance, suggesting a central role in adaptation to high-temperature stress. This study preliminarily confirms that the high-temperature adaptability of *Huso dauricus* is closely related to the structure and composition of its intestinal microbiota, with bacteria such as *Clostridium sensu stricto 1* playing an important role. These findings provide new scientific insights into enhancing fish adaptability to high-temperature stress.

## Simple Summary

As global temperatures rise due to climate change, the survival of fish is increasingly at risk. This study aimed to understand how *Huso dauricus* adapts to high temperatures, focusing on the crucial role of its intestinal microbiota in helping the fish cope with temperature changes. When the fish were exposed to different water temperatures, noticeable changes were observed in the types and proportions of bacteria in their intestines. Over time, the fish adapted to the high temperatures, with one particular type of intestinal bacteria becoming dominant. These findings suggest that the intestinal microbiota of the fish help them adjust to high temperatures. The results of this study are important for understanding how fish can survive in a warming world and may lead to more effective strategies for managing fish populations in the face of climate change.

## 1. Introduction

Global climate warming, accompanied by seasonal increases in water temperature and frequent extreme heatwave events, has resulted in severe, widespread, and irreversible consequences for the survival of aquatic organisms [1]. As ectothermic animals, fish are particularly sensitive to temperature fluctuations. Moreover, intensive aquaculture practices prevent fish from escaping high temperatures through vertical migration, leaving them continuously exposed to high-temperature stress. Prolonged stress can impair fish health and even lead to mortality, severely affecting the economic benefits of aquaculture [1,2,3,4]. Therefore, understanding the mechanisms by which fish respond to and adapt to temperature changes is crucial for developing scientifically sound and effective countermeasures.

Intestinal microbiota, regarded as a “special organ” in fish, are involved in regulating physiological processes such as growth, development, digestion, metabolism, and immune responses. The composition and function of the intestinal microbiota are critical for maintaining fish health and environmental adaptability [5,6,7]. Understanding the changes in intestinal microbiota under high-temperature stress is of significant importance for improving our understanding of fish adaptation to climate warming. Research has shown that temperature fluctuations can reshape the intestinal microbiota in fish. Zhao et al. [8] reported that under high-temperature stress, the diversity of the intestinal microbiota in *Oncorhynchus mykiss* was altered, with significant changes in the relative abundance of Proteobacteria, Firmicutes, and Fusobacteriota. In a study by Kim et al. [9], it was found that elevated water temperature increased the proportion of Firmicutes in the intestinal microbiota of *Chromis notata*, reduced α-diversity, and induced changes in β-diversity. Environmental stress-induced changes in intestinal microbiota can positively affect the physiological functions of the host, thereby enhancing its environmental adaptability [10,11,12,13]. However, under certain conditions, such changes may exacerbate the negative effects of environmental stress [3,4]. Although several studies have observed a correlation between temperature-induced changes in intestinal microbiota and host adaptability [14,15], the underlying mechanisms remain poorly understood.

The kaluga sturgeon (*Huso dauricus*), belonging to the family Acipenseridae, is an economically significant cold-water fish species. Its optimal growth temperature range is between 15 °C and 22 °C, with mortality observed above 25 °C. As one of the oldest fish species on Earth, it has a history of over 200 million years, and its long evolutionary process has endowed it with remarkable environmental adaptability. Therefore, *Huso dauricus* serves as an ideal model for studying the mechanisms of fish adaptation to high-temperature stress. In this study, high-throughput 16S rRNA gene sequencing and bioinformatics were employed to investigate the dynamic changes in the intestinal microbiota under different temperatures (19 °C, 25 °C, 28 °C, and 31 °C). The cessation of rapid mortality in fish is used as an indicator of their adaptation to high-temperature stress. A Venn diagram and heatmap were used to identify the core microbial taxa during this period. Furthermore, co-occurrence network analysis was used to pinpoint keystone species within the core taxa, revealing potential microbial candidates that could enhance fish adaptation to high temperatures. This study preliminarily explores the microbial ecological mechanisms underlying the adaptation of *Huso dauricus* to high-temperature stress. The findings of this study provide a theoretical foundation for the application of microbiome regulation technology to improve the high-temperature adaptability of fish in the future.

## 2. Materials and Methods

### 2.1. Experimental Design and Sample Collection

The experimental F2 generation 5-month-old *Huso dauricus* were sourced from the Hulan Experimental Station of Heilongjiang Fisheries Research Institute, Chinese Academy of Fishery Sciences. A total of 288 healthy fish, exhibiting no visible external injuries and similar body sizes, with an average body weight of (24.76 ± 2.48) g, were evenly distributed into 16 indoor temperature-controlled recirculating aquaculture tanks (60 cm × 40 cm × 50 cm) for temporary rearing. During this period, the light–dark cycle was set at 12 h:12 h, with a water temperature of 19.0 °C, a pH of ~6.9, and a dissolved oxygen concentration of 7.44–7.68 mg/L. Feeding was conducted twice daily at 7:00 and 19:00, with a daily feeding rate equivalent to 3% of the initial body weight for the young fish (Shandong Shengsuo brand sturgeon commercial pellet feed: crude protein ≥ 40%, crude fat ≥ 10%, crude fiber ≤ 6%, crude ash ≤ 18%, moisture ≤ 12%). Daily sludge suction and water exchange were performed before morning feeding, with a water exchange volume of 50% using pre-heated water at the same temperature. After acclimatization for one week, formal experiments were initiated with four groups: a control group maintained at 19 °C and three high-temperature stress groups at 25 °C, 28 °C, and 31 °C, each with four replicates at a stocking density of 18 individuals per tank. The water temperature was gradually adjusted to reach the experimental temperatures at a rate of 1 °C per hour. The management procedures during the experiment were consistent with those during the acclimatization period.

Daily inspections of the tanks were carried out 3 times a day (morning, noon, and evening) to monitor the survival status of *Huso dauricus*. Dead fish were promptly removed and recorded for constructing the survival curve. Sampling was conducted after the rapid mortality phase had ended at each temperature, specifically on day 0 (start of the experiment), day 11 (after rapid mortality ceased at 31 °C), day 26 (after rapid mortality ceased at 28 °C), and day 53 (after rapid mortality ceased at 25 °C). At each sampling point, one fish was randomly selected from each tank in each group, 4 h after feeding, totaling four fish per group. The fish were anesthetized with 40 mg/L MS-222 (Sigma-Aldrich, Beijing, China) and then dissected on a clean ice surface for sampling. Intestinal contents were carefully extruded from the foregut to the hindgut using sterile forceps, thoroughly mixed in sterile culture dishes, and transferred into a 1.5 mL sterile centrifuge tube, with each fish serving as an independent sample. The samples were flash-frozen in liquid nitrogen and then stored at −80 °C for microbiome analysis.

### 2.2. DNA Extraction and Bacterial 16S rRNA Gene Sequencing

The total bacterial DNA from the intestinal content samples was isolated with the HiPure Stool DNA Kit (Magen Biotech Inc., Guangzhou, China) following the provided protocol. The DNA concentration and quality were assessed using a NanoDrop™ 2000 spectrophotometer (NanoDrop Technologies, LLC, Wilmington, DE, USA). All DNA samples met the required standards for PCR amplification. The amplification of the V3-V4 region of the 16S rRNA gene was conducted using the primers 338F (5′-ACTCCTACGGGAGGCAGCA-3′) and 806R (5′-GGACTACHVGGGTWTCTAAT-3′). PCR was performed with a 30 μL reaction mixture comprising 15 μL of 2× Gflex Buffer, 2 μL of dNTPs (2.5 mM), 1 μL each of the forward and reverse primers (5 μM), 0.6 μL of Tks Gflex™ DNA Polymerase (1.25 U/μL), and 50 ng of the DNA template. The amplification was executed on an ABI GeneAmp^®^ 9700 (ABI, Carlsbad, CA, USA) with the following thermal profile: initial denaturation at 94 °C for 5 min, followed by 26 cycles of denaturation at 94 °C for 30 s, annealing at 56 °C for 30 s, and extension at 72 °C for 20 s, ending with a final extension at 72 °C for 5 min. Three independent PCR reactions were conducted for each sample. The quality of the PCR products was evaluated using agarose gel electrophoresis. PCR products that were successful were purified with magnetic beads. The purified PCR products served as templates for a second round of amplification. The second PCR round employed the same reaction mix and conditions as the first, but with only seven cycles. After the second round of PCR, the products were re-evaluated using agarose gel electrophoresis and purified again with magnetic beads. The concentration of the purified products was measured with a Qubit 2.0 fluorometer (Life Technologies, Carlsbad, CA, USA). The samples were combined in equimolar amounts according to the quantified concentrations of the PCR products. The combined samples were subsequently sequenced using the NovaSeq 6000 platform (Illumina, Inc., San Diego, CA, USA).

### 2.3. Processing of Illumina Sequencing Data

Raw 16S rRNA amplicon sequences were analyzed using QIIME2 (2020.11) [16]. The DADA2 pipeline was employed for denoising, which produced a table of amplicon sequence variants (ASVs) [17]. Representative sequences for each ASV were chosen using the default settings and taxonomically assigned via the SILVA 138 database [18]. For consistency across samples, the ASV abundance table was rarefied to the lowest sequence depth of 65,708.

### 2.4. Statistical Analyses

The survival curve was plotted using GraphPad Prism 8 (GraphPad Software Inc., San Diego, CA, USA) software. Taxonomic compositions of microbiota were determined using the QIIME command “*summarize_taxa_through_plots.py*”. Alpha diversity was assessed using the species richness index (observed species), which was calculated with “*alpha_diversity.py*” and “*collate_alpha.py*”. Beta diversity was assessed using Bray–Curtis distance metrics, which were calculated with the command “*beta_diversity.py*”. Principal Coordinates Analysis (PCoA) based on the Bray–Curtis distance was used to visualize differences in the microbiota composition among groups. Clustering analysis of the microbiota composition among groups based on the Bray–Curtis distance was performed using the unweighted pair-group method with the arithmetic mean (UPGMA) from the R package “phangorn”. Microbiota structure differences were assessed using ADONIS (Permutational Multivariate Analysis of Variance, PERMANOVA) from the R package “vegan”. One-way Analysis of Variance (ANOVA) was performed using the R function “aov”, followed by Tukey’s post hoc test from the “multcomp” package. Relative abundance differences were analyzed using Linear Discriminant Analysis (LDA) Effect Size (LEfSe) (LDA score > 3.0) on the Galaxy tool (Galaxy server 2.0; http://galaxy.biobakery.org/; accessed: 22 August 2024) [19]. ASVs with a relative abundance greater than 1% in at least one sample were selected, and the Spearman correlation coefficients between all ASV pairs were calculated. Pairs with R > 0.6 and *p* < 0.05 were retained to construct a co-occurrence network. Spearman correlations were calculated using the R package “Hmisc”. The co-occurrence network was visualized by Gephi 0.10 [20]. Based on the network analysis results, ASVs that rank in the top five for all of the following metrics—relative abundance, degree, closeness centrality, harmonic closeness centrality, betweenness centrality, authority, hub, pagerank, and eigencentrality—are defined as the “keystone” of the network. Data visualizations were created using the R package “ggplot2”.

## 3. Results

### 3.1. Survival Status Under Different Temperatures

**Figure 1:**
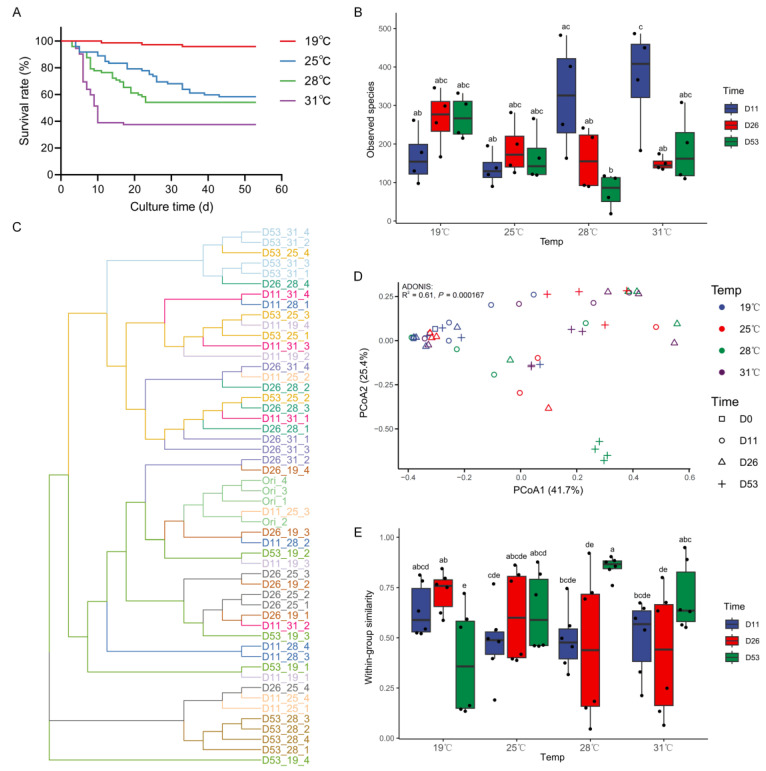
(**A**) Survival curve, (**B**) species richness index (observed species) of intestinal microbiota, (**C**) UPGMA clustering of intestinal microbiota based on the Bray–Curtis distance, (**D**) PCoA of intestinal microbiota based on the Bray–Curtis distance, (**E**) within-group similarities of intestinal microbiota based on the Bray–Curtis distance under different temperatures and times in *Huso dauricus*. Different letters indicate significant difference (*p* < 0.05). Dx_y_z represents the z-th fish on day x under temperature y.

High-temperature stress caused a large number of deaths in *Huso dauricus*, with the mortality rate increasing as the temperature rose (Figure 1A). The cumulative mortality over 53 days at 19 °C, 25 °C, 28 °C, and 31 °C was 0.42%, 41.6%, 45.8%, and 62.5%, respectively. Notably, all the deaths occurred during the early stages of high-temperature stress and became more rapid as the temperature increased. Rapid mortality at 25 °C, 28 °C, and 31 °C was concentrated within the first 43 days, 25 days, and 10 days, respectively.

### 3.2. Diversity of Intestinal Microbiota Under Different Temperatures

The diversity of intestinal microbiota in *Huso dauricus* showed significant differences under different temperatures (Figure 1B–D). On day 11, the species richness index at 31 °C was significantly higher than that at 19 °C and 25 °C (*p* < 0.05). However, after day 26, no significant differences in the species richness index were found across temperatures (*p* > 0.05). The species richness index of intestinal microbiota decreased over time at 28 °C and 31 °C, while it remained stable at 19 °C and 25 °C (Figure 1B). UPGMA clustering analysis, PCoA, and ADONIS results all showed that on day 11, there were no significant differences in the intestinal microbiota structure among the different temperatures (*p* > 0.05). However, starting on day 26, the differences in the intestinal microbiota structure among the temperatures gradually increased, reaching a significant level by day 53 (*p* < 0.05). At 19 °C, the intestinal microbiota structure did not change significantly over time, whereas at 25 °C, 28 °C, and 31 °C, the intestinal microbiota structure changed significantly after day 26 (*p* < 0.05, Figure 1C,D, Table S1). Additionally, the within-group similarity of the intestinal microbiota structure under high-temperature stress showed a trend of initially decreasing (0–11 days) and then increasing (11–53 days) (Figure 1D,E).

### 3.3. Dominant Bacterial Composition of Intestinal Microbiota Under Different Temperatures

**Figure 2:**
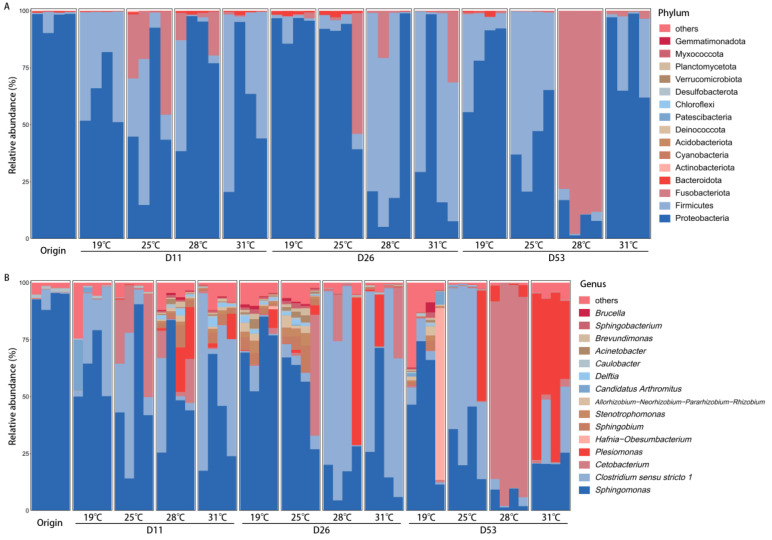
Dominant bacterial composition of intestinal microbiota under different temperatures and times in *Huso dauricus* (top 15 relative abundances): (**A**) phylum level; (**B**) genus level. Dx represents day x.

The composition of the intestinal microbiota in *Huso dauricus* showed significant differences under different temperatures (Figure 2). At the phylum level, the dominant bacterial phyla in the intestines across the different temperatures were similar: on day 11, Proteobacteria and Firmicutes were the dominant phyla at both 19 °C and 31 °C, while Proteobacteria, Firmicutes, and Fusobacteriota were dominant at 25 °C and 28 °C. On day 26, Proteobacteria was the dominant phylum in the intestines at both 19 °C and 25 °C, while at 28 °C and 31 °C, Proteobacteria and Firmicutes were dominant. On day 53, the dominant phyla at 19 °C, 25 °C, and 31 °C were Proteobacteria and Firmicutes, whereas Fusobacteriota was dominant at 28 °C. At the genus level, the differences in the dominant bacterial genera across temperatures were more pronounced: on day 11, the dominant genera at 19 °C and 31 °C were *Sphingomonas* and *Clostridium sensu stricto 1*, while at 25 °C, *Sphingomonas*, *Clostridium sensu stricto 1*, and *Cetobacterium* were dominant, and at 28 °C, *Sphingomonas* was dominant. On day 26, *Sphingomonas* was the dominant genus at both 19 °C and 25 °C, while at 28 °C and 31 °C, *Sphingomonas* and *Clostridium sensu stricto 1* were dominant. On day 53, the dominant genera showed distinct differences across temperatures: *Sphingomonas* was dominant at 19 °C, *Sphingomonas* and *Clostridium sensu stricto 1* were dominant at 25° C, *Cetobacterium* was dominant at 28 °C, and *Sphingomonas* and *Plesiomonas* were dominant at 31 °C.

### 3.4. Differential Bacterial Composition of Intestinal Microbiota Under Different Temperatures

**Figure 3:**
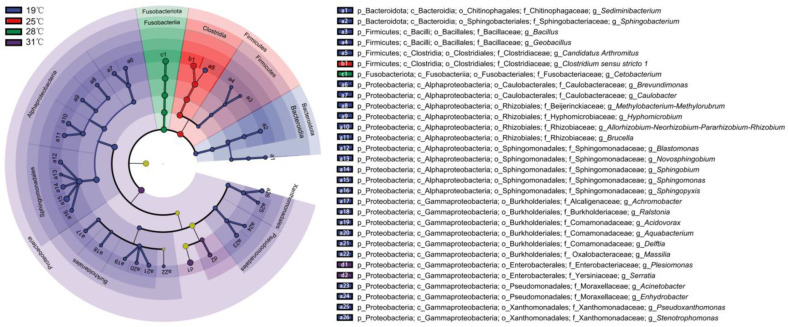
LefSE of the intestinal microbiota in *Huso dauricus* on day 53 under different temperatures at the genus level with an LDA score > 3.

The differential intestinal bacteria on day 53 under different temperatures were selected based on LefSE (Figure 3 and Figure S1). A total of 30 bacterial genera showed significant differences (*p* < 0.05). Among them, the largest number of differential genera were observed at 19 °C, with 26 genera, primarily belonging to Proteobacteria, Firmicutes, and Bacteroidota. At 25 °C, *Clostridium sensu stricto 1* was the dominant genus (relative abundance 33.8–78.6%). At 28 °C, the dominant genus was *Cetobacterium* (78.0–97.9%). At 31 °C, two dominant genera were identified: *Plesiomonas* (34.3–74.4%) and *Serratia* (0.3–0.5%).

### 3.5. Relationship Between Intestinal Microbiota and High-Temperature Adaptability

**Figure 4:**
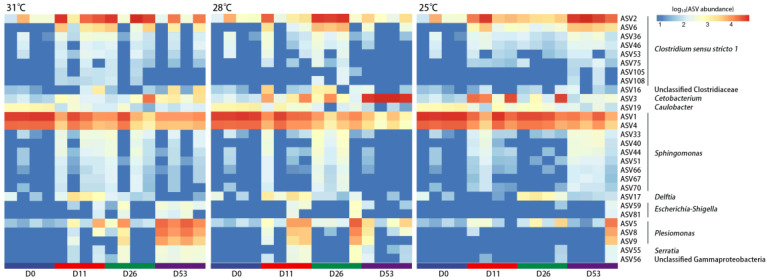
Heatmap of shared ASVs (D11_31, D26_28, D53_25) in the intestinal microbiota of *Huso dauricus* under different temperatures and times.

*Huso dauricus* showed adaptation to high-temperature stress, as no further mortality occurred after a period of time (Figure 1A). Meanwhile, the intestinal microbiota at the time when rapid mortality ceased (D11_31, D26_28, D53_25) under different high temperatures showed a highly similar structure and composition (Figure 1C,D, Figure 2). A Venn diagram was used to identify the shared bacteria, revealing 28 shared ASVs and 136 non-shared ASVs (Figure S2). These shared ASVs primarily belong to *Clostridium sensu stricto 1*, *Cetobacterium*, *Sphingomonas*, and *Plesiomonas* (Figure 4). Among them, ASV2 of *Clostridium sensu stricto 1* exhibited a higher relative abundance during days 11–53 at 31 °C, days 26–53 at 28 °C, and day 53 at 25 °C, which corresponds to the period when *Huso dauricus* adapted to high temperatures.

**Figure 5:**
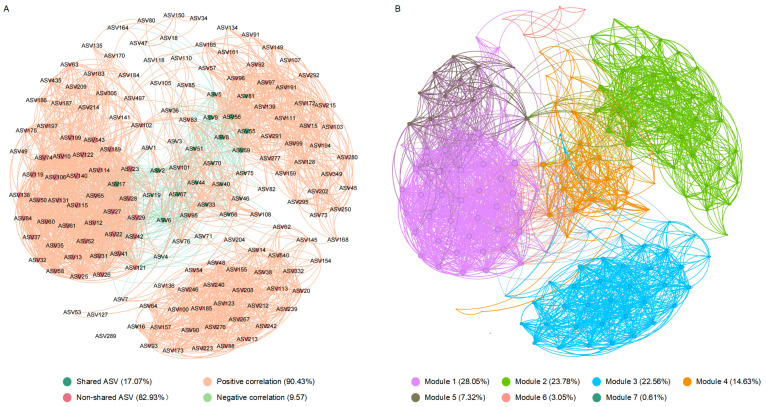
Co-occurrence networks of the intestinal microbiota in *Huso dauricus* (D11_31, D26_28, D53_25, relative abundance > 0.1% at least in one sample). The nodes are colored based on whether they are shared ASVs (**A**) and modules (**B**), respectively. The size of each node is proportional to the degree.

The interactions among intestinal bacteria were inferred based on co-occurrence network analysis (Figure 5). The results showed that the shared ASVs were primarily clustered in the same module (Module 4), with close interactions observed among them. Additionally, network topological parameters such as the degree, closeness centrality, harmonic closeness centrality, and betweenness centrality all indicated that ASV2 is a crucial central node, serving as the “keystone” of the co-occurrence network (Figure 5, Table S2).

## 4. Discussion

### 4.1. Different Temperatures Shape Intestinal Microbiota with Distinct Characteristics

The plasticity of the intestinal microbiota is an important factor determining the phenotypic plasticity of vertebrates, playing a significant role in their adaptation to and response to rapid environmental changes [10,21]. This study investigated the dynamics of the intestinal microbiota in *Huso dauricus* under different temperatures over time. The results showed that different temperatures shaped intestinal microbiota with distinct characteristics. Under high-temperature stress, the species richness index of the intestinal microbiota significantly increased, first rising and then decreasing over time. This may be due to the disruption of the microbiota balance caused by temperature changes, leading to a transient increase in certain bacteria, thereby increasing the species richness index. As time progressed, the intestinal microbiota gradually adapted and became stable, leading to a decrease in the species richness index and a return to normal levels [22]. This may also explain why variations in α-diversity have been observed in different studies, with some showing an increase [3] while others report a decrease [4,9]. Such discrepancies are likely due to differences in sampling time points, as different periods may reflect distinct stages in the response of the intestinal microbiota to high-temperature stress. It was also observed that the structure of the intestinal microbiota remained relatively stable over time under normal temperature conditions, whereas significant changes were noted under high-temperature stress. Additionally, the differences in the microbiota structure over time became increasingly significant under different temperature conditions, with a gradual reduction in intra-group variation. These results suggest that high temperatures induce changes in the intestinal microbiota and that different temperatures shape distinct microbiota structures. These findings are consistent with those of Zhang et al. [21] on *Cyprinus carpio* and Hassenrück et al. [23] on *Chanos chanos*. In addition, the same pattern has also been observed in studies of other species, such as *Xenopus tropicalis* [24] and *Anas platyrhynchos* [25].

In this study, notable differences were found in the dominant bacterial compositions under different temperature conditions, and these differences became more pronounced over time. Under high temperatures, the relative abundance of *Sphingomonas* decreased significantly, while the relative abundance of *Clostridium sensu stricto 1*, *Cetobacterium*, and *Plesiomonas* increased significantly. *Sphingomonas* is one of the core microbiota in the intestine of sturgeon [26], playing a crucial role in maintaining fish health [27]. High-temperature stress disrupts the original microbial balance, leading to a decrease in the abundance of certain indigenous bacteria [28]. *Clostridium sensu stricto 1* is also a core microbiota of sturgeon [29], involved in the breakdown of carbohydrates and proteins and exhibiting probiotic functions that promote host digestion [30]. Under high-temperature stress, fish experience increased energy expenditure [31], and the high relative abundance of *Clostridium sensu stricto 1* may be key to maintaining the energy metabolism balance in fish. *Cetobacterium* is known to play an important role in carbohydrate degradation and is commonly found in the intestines of herbivorous and omnivorous fish species [32,33]. It has been shown to effectively improve intestinal health and enhance disease resistance in fish [34,35]. The high relative abundance of *Cetobacterium* may be a crucial factor in maintaining fish survival under high-temperature stress. *Plesiomonas* is also one of the core microbiota of sturgeon [36] and is typically considered an opportunistic pathogen [37], which may partially explain why the mortality rate of sturgeon is highest at 31 °C. In summary, through the study of dynamic changes in the intestinal microbiota under different temperature conditions, it has been demonstrated that the intestinal microbiota of *Huso dauricus* is highly plastic, and this plasticity likely plays a vital role in adapting to environmental changes.

### 4.2. Specific Intestinal Bacterial Taxa Are Associated with the Development of High-Temperature Adaptation

The intestinal microbiota is closely related to the environmental adaptability of its host, with the presence of specific bacteria playing a critical role [22]. Studies on *Penaeus vannamei* [38] and *Drosophila melanogaster* [39] have demonstrated that specific intestinal bacteria can influence environmental adaptability in the host. In this study, the intestinal microbiota structure and composition of *Huso dauricus* under different high-temperature stresses showed striking similarity when rapid mortality ceased, with *Clostridium sensu stricto 1* dominating both in terms of composition and relative abundance. *Clostridium sensu stricto 1* is known to possess digestive-promoting functions [30], and its increased abundance may help *Huso dauricus* compensate for energy deficits under high-temperature stress, ensuring survival under conditions of increased energy demand. A study on mice also confirmed that *Clostridium* can enhance energy absorption and promote fat generation in the host [40]. Co-occurrence network analysis provides a comprehensive perspective for studying the interactions among intestinal bacteria in aquatic animals [41,42]. In this study, the microbial co-occurrence network of *Huso dauricus* under high-temperature stress was divided into seven modules, with shared ASVs primarily clustering in the same module. Bacteria within the same module are often more closely related in terms of survival or function [43], suggesting that the shared ASVs may jointly contribute to the adaptation process of high temperatures. Furthermore, both the heatmap and network topological parameters indicated that ASV2 of *Clostridium sensu stricto 1* is the “keystone” of the intestinal microbiota under high-temperature stress, and changes in its abundance may directly influence the high-temperature adaptability. This finding was also confirmed in two studies on pigs, where hosts with a higher abundance of *Clostridium sensu stricto 1* in the intestine exhibited better high-temperature adaptability [44,45]. In summary, this study preliminarily confirms that the high-temperature adaptability of *Huso dauricus* is closely related to the structure and composition of its intestinal microbiota, with bacteria such as *Clostridium sensu stricto 1* playing an important role. In the future, the abundance of *Clostridium sensu stricto 1* in the intestine could be increased through microbiota modulation techniques, thereby enhancing the adaptability of the fish to high-temperature stress. This approach could ultimately lead to more sustainable and efficient aquaculture practices, ensuring fish welfare and optimizing production in the face of climate change challenges.

## 5. Conclusions

This study demonstrates that temperature variation can reshape the structure and composition of the intestinal microbiota in *Huso dauricus*, with distinct characteristics of the microbiota formed under different temperature conditions. The intestinal microbiota is closely associated with the development of high-temperature adaptation in *Huso dauricus*, with intestinal bacteria, primarily *Clostridium sensu stricto 1*, playing a key role. These findings provide new insights into the mechanisms underlying the development of high-temperature adaptation in *Huso dauricus* and offer a theoretical basis for the use of microbiota-based intervention strategies to enhance high-temperature adaptability in fish.

## Data Availability

All raw sequencing data have been deposited in the Genome Sequence Archive at the National Genomics Data Center (https://bigd.big.ac.cn/gsa; accessed: 28 August 2024), Beijing Institute of Genomics (China National Center for Bioinformation), Chinese Academy of Sciences, with the accession number CRA020801.

## Supplementary Materials

The following supporting information can be downloaded at: https://www.mdpi.com/article/10.3390/biology13121045/s1, Figure S1: LDA scores distribution of the intestinal microbiota in *Huso dauricus* on day 53 under different temperatures at the genus level with an LDA score > 3; Figure S2: Venn diagram at the ASV level of intestinal microbiota in *Huso dauricus* for D11_31, D26_28, and D53_25; Table S1: ADONIS of intestinal microbiota in *Huso dauricus* under different temperatures and times based on Bray–Curtis distance; Table S2: Co-occurrence network topological parameters of shared ASVs (D11_31, D26_28, D53_25).
