# Supplementary material for: Exploring the Adaptation Process of Huso dauricus to High Temperatures Based on Changes in Intestinal Microbiota
Source: Biology (Basel). 2024 Dec 13;13(12):1045. doi: 10.3390/biology13121045 (PMC11672952; doi:10.3390/biology13121045)
Supplement: Supplementary file 1 [file biology-13-01045-s001.zip › biology-3347868-supplementary.pdf]

Table S1. ADONIS of intestinal microbiota in *Huso dauricus* under different temperatures and times based on Bray-Curtis distance.

Pairs	ADONIS	
	R^2	*P*
Ori vs D11_19	0.463	**0.031**
Ori vs D11_25	0.494	0.062
Ori vs D11_28	0.465	**0.028**
Ori vs D11_31	0.586	**0.026**
Ori vs D26_19	0.473	**0.027**
Ori vs D26_25	0.502	**0.028**
Ori vs D26_28	0.628	**0.033**
Ori vs D26_31	0.558	0.065
Ori vs D53_19	0.296	0.032
Ori vs D53_25	0.775	**0.029**
Ori vs D53_28	0.976	**0.028**
Ori vs D53_31	0.865	**0.028**
D11_19 vs D11_25	0.206	0.260
D11_19 vs D11_28	0.244	0.171
D11_19 vs D11_31	0.235	0.176
D11_19 vs D26_19	0.323	0.058
D11_19 vs D26_25	0.307	**0.032**
D11_19 vs D26_28	0.379	**0.081**
D11_19 vs D26_31	0.276	0.144
D11_19 vs D53_19	0.159	0.260
D11_19 vs D53_25	0.429	**0.028**
D11_19 vs D53_28	0.863	**0.032**
D11_19 vs D53_31	0.677	**0.032**
D11_25 vs D11_28	0.189	0.259
D11_25 vs D11_31	0.214	0.231
D11_25 vs D26_19	0.408	0.059
D11_25 vs D26_25	0.260	0.059
D11_25 vs D26_28	0.252	0.085
D11_25 vs D26_31	0.159	0.403
D11_25 vs D53_19	0.219	0.140
D11_25 vs D53_25	0.302	0.113

D11_25 vs D53_28	0.613	**0.026**
D11_25 vs D53_31	0.545	**0.029**
D11_28 vs D11_31	0.180	0.278
D11_28 vs D26_19	0.195	0.200
D11_28 vs D26_25	0.107	0.659
D11_28 vs D26_28	0.341	0.056
D11_28 vs D26_31	0.282	0.177
D11_28 vs D53_19	0.139	0.515
D11_28 vs D53_25	0.406	0.080
D11_28 vs D53_28	0.726	**0.032**
D11_28 vs D53_31	0.470	**0.029**
D11_31 vs D26_19	0.409	0.053
D11_31 vs D26_25	0.305	0.119
D11_31 vs D26_28	0.152	0.297
D11_31 vs D26_31	0.086	0.519
D11_31 vs D53_19	0.226	0.163
D11_31 vs D53_25	0.134	0.461
D11_31 vs D53_28	0.762	**0.030**
D11_31 vs D53_31	0.516	**0.034**
D26_19 vs D26_25	0.156	0.256
D26_19 vs D26_28	0.558	**0.027**
D26_19 vs D26_31	0.480	0.057
D26_19 vs D53_19	0.158	0.309
D26_19 vs D53_25	0.676	**0.028**
D26_19 vs D53_28	0.911	**0.029**
D26_19 vs D53_31	0.769	**0.029**
D26_25 vs D26_28	0.461	0.056
D26_25 vs D26_31	0.381	0.062
D26_25 vs D53_19	0.139	0.460
D26_25 vs D53_25	0.558	**0.027**
D26_25 vs D53_28	0.782	**0.026**
D26_25 vs D53_31	0.656	**0.031**
D26_28 vs D26_31	0.029	0.893
D26_28 vs D53_19	0.349	0.061

D26_28 vs D53_25	0.018	0.943
D26_28 vs D53_28	0.653	**0.027**
D26_28 vs D53_31	0.375	0.144
D26_31 vs D53_19	0.287	0.143
D26_31 vs D53_25	0.009	0.938
D26_31 vs D53_28	0.638	**0.027**
D26_31 vs D53_31	0.435	0.090
D53_19 vs D53_25	0.398	**0.029**
D53_19 vs D53_28	0.665	**0.027**
D53_19 vs D53_31	0.493	**0.025**
D53_25 vs D53_28	0.844	**0.026**
D53_25 vs D53_31	0.572	0.056
D53_28 vs D53_31	0.880	**0.028**

Note: Bolded values indicating significant differences. Dx_y represents day x under temperature y.

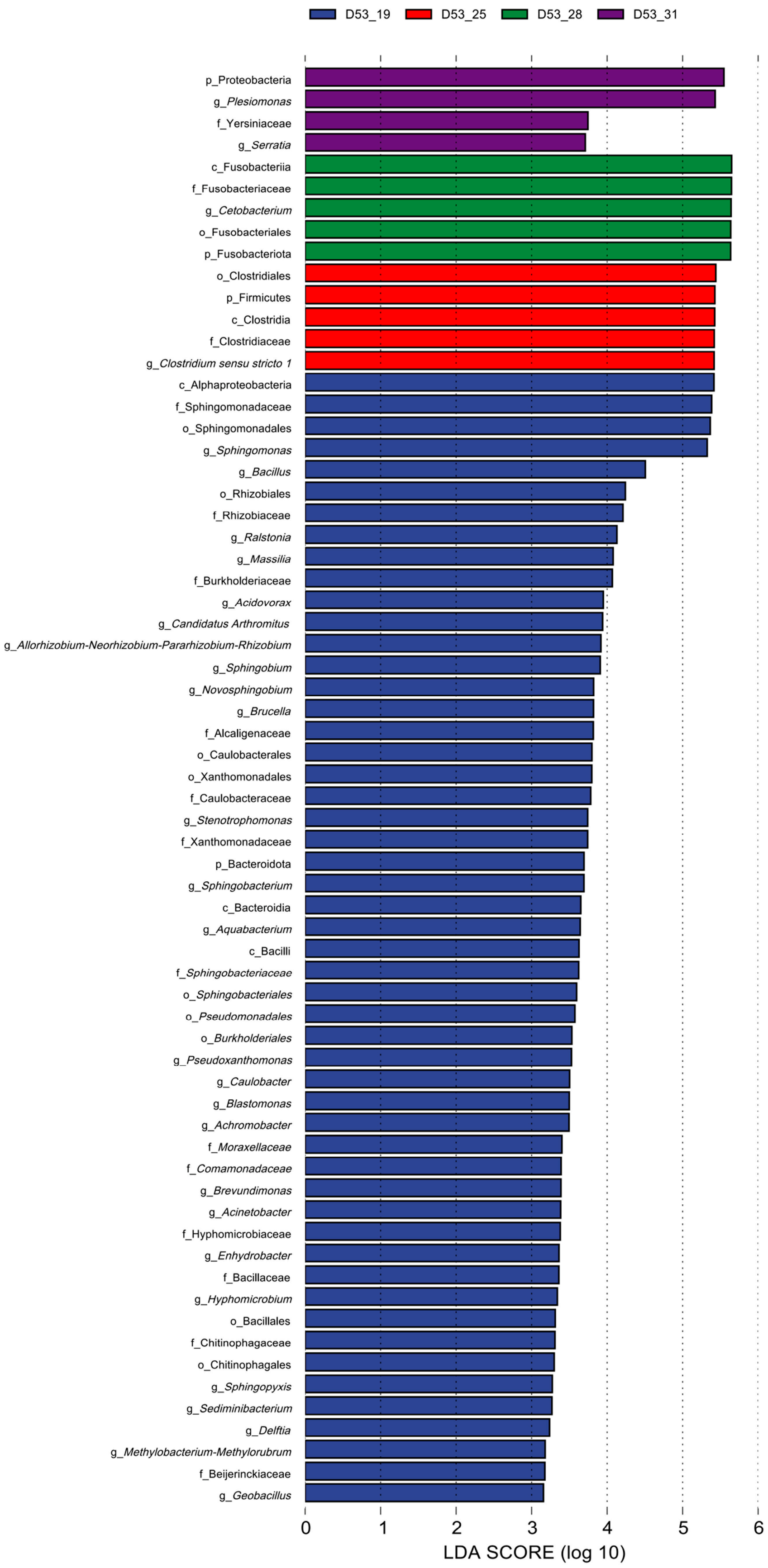

Figure S1. LDA scores distribution of the intestinal microbiota in *Huso dauricus* on day 53 under different temperatures at the genus level with an LDA score > 3.

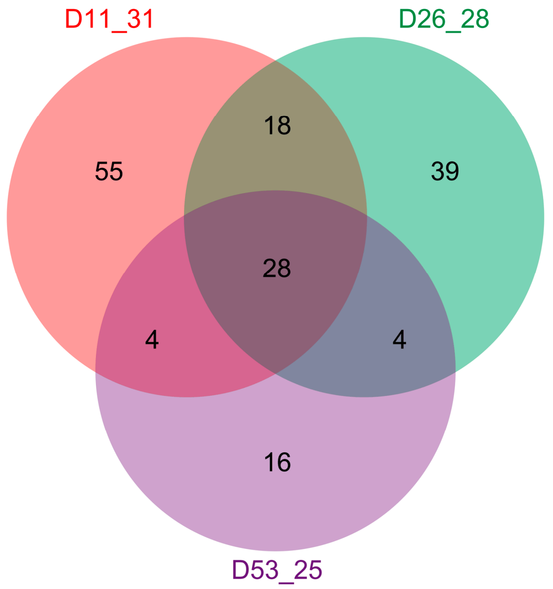

Figure S2. Venn diagram at the ASV level of intestinal microbiota in *Huso dauricus* for D11_31, D26_28, and D53_25.

Table S2. Co-occurrence network topological parameters of shared ASVs (D11_31, D26_28, and D53_25).

ASV ID	Degree	Closness centrality	Harmonic closness centrality	Betweeness centrality	Authority	Hub	Pageranks	Modularity class	Eigencentrality
ASV2	42	0.440	0.550	986.916	0.093	0.093	0.007	4	0.644
ASV6	35	0.430	0.526	334.093	0.085	0.085	0.006	4	0.568
ASV56	44	0.411	0.548	390.200	0.027	0.027	0.008	4	0.341
ASV55	44	0.411	0.548	390.200	0.027	0.027	0.008	6	0.341
ASV59	44	0.411	0.548	390.200	0.027	0.027	0.008	6	0.341
ASV44	31	0.407	0.509	158.165	0.053	0.053	0.005	4	0.398
ASV67	36	0.435	0.533	361.887	0.069	0.069	0.006	4	0.495
ASV70	26	0.401	0.493	94.520	0.037	0.037	0.005	4	0.304
ASV1	13	0.379	0.430	101.759	0.020	0.020	0.003	1	0.122
ASV4	13	0.379	0.430	101.759	0.020	0.020	0.003	1	0.122
ASV19	23	0.391	0.465	290.534	0.073	0.073	0.004	1	0.428
ASV5	34	0.387	0.501	529.369	0.017	0.017	0.007	6	0.238
ASV8	40	0.407	0.535	303.014	0.026	0.026	0.007	6	0.322
ASV9	39	0.390	0.516	394.553	0.024	0.024	0.007	6	0.308
ASV40	28	0.387	0.489	65.999	0.037	0.037	0.005	4	0.308
ASV81	31	0.373	0.481	78.647	0.010	0.010	0.006	6	0.208
ASV51	29	0.387	0.491	76.465	0.041	0.041	0.005	4	0.333
ASV75	21	0.372	0.459	71.138	0.016	0.016	0.004	4	0.171
ASV66	25	0.381	0.477	33.812	0.028	0.028	0.005	4	0.251
ASV3	10	0.333	0.370	156.950	0.002	0.002	0.003	3	0.021
ASV17	47	0.420	0.546	99.449	0.162	0.162	0.007	1	0.974
ASV16	23	0.212	0.310	0.000	0.000	0.000	0.004	3	0.134
ASV33	31	0.415	0.508	170.545	0.053	0.053	0.005	4	0.398
ASV46	23	0.379	0.471	182.043	0.024	0.024	0.004	4	0.220
ASV108	14	0.315	0.376	0.565	0.012	0.012	0.003	4	0.110
ASV36	21	0.352	0.434	150.210	0.011	0.011	0.004	4	0.135
ASV53	4	0.245	0.274	1.083	0.000	0.000	0.001	4	0.004
ASV105	6	0.269	0.310	0.367	0.001	0.001	0.002	4	0.014
